# A Rare Variation of the Heterotaxy Syndrome

**DOI:** 10.1155/2012/840453

**Published:** 2012-06-28

**Authors:** Alper Dilli, Salih Sinan Gultekin, Umit Yasar Ayaz, Hatice Kaplanoglu, Baki Hekimoglu

**Affiliations:** ^1^Department of Radiology, Diskapi Yildirim Beyazit Training and Research Hospital, Ministry of Health, O6110 Ankara, Turkey; ^2^Department of Nuclear Medicine, Diskapi Yildirim Beyazit Training and Research Hospital, Ministry of Health, O6110 Ankara, Turkey; ^3^Department of Radiology, Mersin Women's and Children's Hospital, Ministry of Health, 33240 Mersin, Turkey

## Abstract

Heterotaxy syndrome is a rare, complex, and confusing type of the situs anomalies. It is not possible to estimate the degree of lateralization, isomerism, and rotational variation in these types of cases. Heart and abdominal organ anatomy is specific to the individual, and it should be defined specifically on the basis of each case due to possible cardiac and extracardiac surgical interventions in patients with heterotaxy syndrome. Here, we present our findings obtained from a 58-year-old female patient with heterotaxy syndrome. The main components of this rare variation consist of right-hand-sided aorta, aortic arc, cardiac apex, gall bladder and left-hand-sided inferior vena cava, stomach, and spleen (polysplenia, 3 foci) according to the midline. Besides, the components include left-dominant liver, right-hand-sided large intestines, and left-hand-sided small intestines.

## 1. Introduction

Situs anomalies are rare and complex entities which may be partial or total. A basic terminology has been defined for situs anomalies by some authors [1–3]. The term situs is used to describe the positions of the  heart and abdominal viscera by midline. Situs solitus represents the normal position, with left-hand-sided aorta, cardiac apex, spleen, stomach, and right-hand-sided liver and inferior vena cava. In situs inversus, abdominal viscera are in translocated position as the mirror image of situs solitus. The position of the cardiac apex in situs inversus may be in the form of dextrocardia or levocardia. The condition which is called situs ambiguous or heterotaxy syndrome is less common, and it  represents an intermediate degree of visceral malposition, dismorphism, and atrial disposition which are incongruent with situs solitus or inversus [1, 4–6]. Immature or duplicated unilateral structures [1, 7], left-dominant or right-dominant liver at the midline [2], gastrointestinal rotation anomalies [3], or the presence of complicated atrial morphology and congenital heart diseases [8] may be seen. Heterotaxy syndromes can be evaluated further in two main subgroups, including right-sided isomerism with asplenia and left-sided isomerism with polysplenia [4–6]. In the form of right-sided isomerism with asplenia, the patients have more severe congenital heart diseases and lateralization defects. There is no single type specific condition or particular pathognomonic finding for left-sided isomerism with polysplenia. Ambiguous or midline positions for abdominal organs are likely to be one of the forms of settlement. The majority of the patients (up to 90%) have less severe congenital heart diseases [1–3, 5].

## 2. Case Report

**Figure 1:**
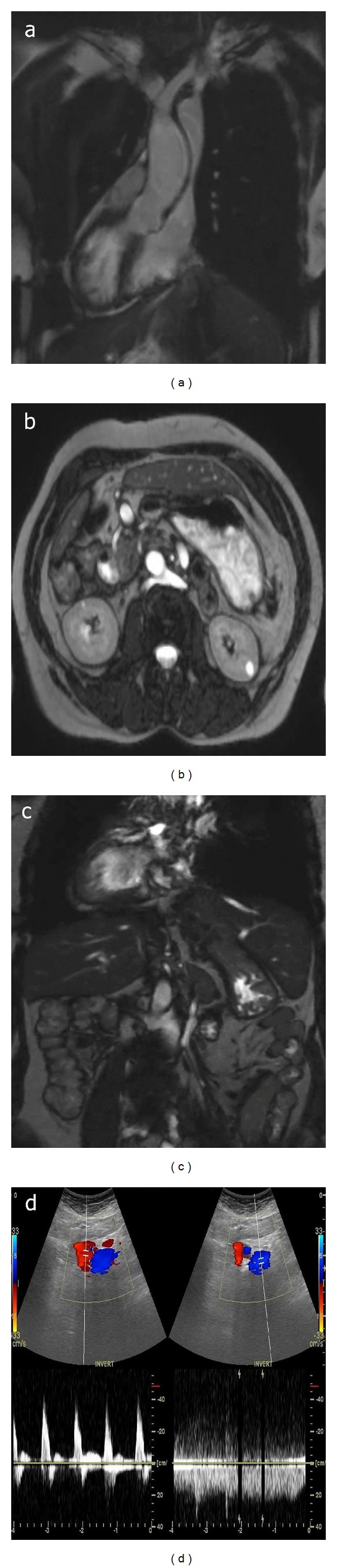
BTFE-BH SENSE sequence on MRI and CDUS. (a) On coronal MRI, right-sided apex of the heart (dextrocardia), aorta in the middle, the pulmonary artery at the right of the aorta, and the superior vena cava at the left of the aorta are observed. (b) and (c) Axial and coronal MRI images show right-sided aorta, left-sided inferior vena cava, and retroaortic right renal vein. (d) In CDUS, current and spectrum images for the aorta at the right-hand side and the inferior vena cava at the left-hand side are shown.

**Figure 2:**
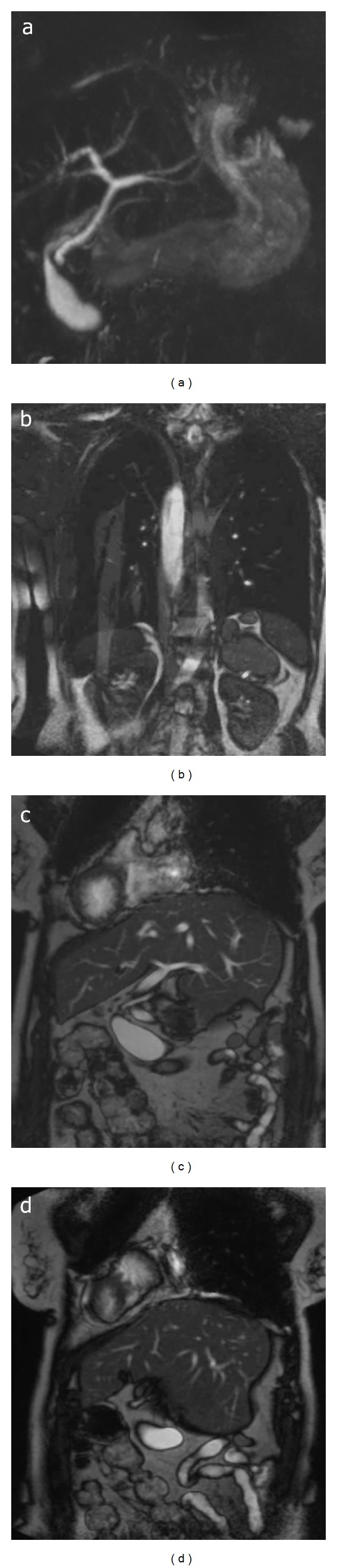
MRCP and BTFE-BH SENSE sequence on MRI. (a) MRCP imaging shows that the liver is left dominant in the midline, the common hepatic duct and common bile duct extend from the left superolateral to the inferomedial, and the pancreas is in normal location. (b) On coronal BTFE-BH SENSE sequence on MRI, the spleen is seen with three foci at the left below the liver congruent with polysplenism. (c) and (d) On coronal BTFE-BH SENSE sequence on MRI, the liver is left dominantly located at the both sides of the midline and the portal vein drains into the liver leading from the left. The intestines seem to be congruent with malrotation, and, predominantly, large intestines on the right-hand side and small intestines on the left-hand side according to the midline.

A 58-year-old female had lumbar pain and shortness of breath at the time of admission. Medical history revealed the patient being under medical treatment for chronic obstructive lung disease. A slight limitation in lumbar movements was determined by physical examination. Routine laboratory tests were normal. Further radiological evaluation included ultrasonography (US), color Doppler ultrasonography (CDUS), thoracoabdominal magnetic resonance imaging (MRI), and magnetic resonance cholangiopancreatography (MRCP). The balanced turbo field echo (BTFE-BH SENSE) sequence on MRI (Figures 1 and 2) revealed following findings: the aorta, aortic arc, and apex of the heart were located on the right-hand side, and the inferior vena cava, stomach, and spleen were located on the left-hand side according to the midline. The liver was located in the midline, but it was left-dominant liver. The position of the gallbladder was right-hand sided under the liver. The pancreas was located on its normal position in the midline. The spleen had three foci (polysplenia) and was in a settled position on the left-hand side under the liver. The right renal vein which was retroaortic has passed posterior to the aorta and drained into the inferior vena cava. The small and large intestines seemed to have some degree of malrotation. It was observed that the  large and small intestines were predominantly settled in lower right region and upper left region of the abdomen, respectively. Color Doppler ultrasonography showed that the inferior vena cava and aorta flows were on the opposite side of the normal location (Figure 1(d)). On MRCP, the liver was observed to be left dominant located, and common bile duct was lying towards the inferomedial from the left-hand side.

## 3. Discussion

The concept of  the lateralization in the human body has a special importance in terms of organs and systems, especially for cardiovascular, pulmonary, and gastrointestinal systems developed around the embryonic midline. The control mechanism of somatic asymmetry in humans is unclear. Asymmetric or unilateral organs develop from the embryologic midline structures [3, 5]. Minor changes in “embryonic body curvatures” in early embryonic period may help the explanation of the varied anatomic spectrum in heterotaxy syndromes [1]. This condition probably arises from a defect in the lateralization, and deformation of the embryonic laterality causes development disorder in asymmetric organs. Viscerovascular regulation seems to occur sporadically and in a wide spectrum for the right or left lateralization when the solitus control is missing [5]. Although some of the situs ambiguous cases are sporadic, autosomal dominant and autosomal recessive hereditary patterns and X-linked inheritance pattern have also been described [1–3, 5]. Today, genetic research supports mostly a multifactorial hereditary model [4].

Although situs ambiguous cases are rarely seen, various anomalies in varying degrees related to cardiopulmonary system and intra-abdominal or vascular structures are observed in these cases [1–9]. In cardiovascular system, biventricular heart and large atrioventricular defects may be seen. Complex cardiac lesions are observed more in patients with right-sided isomerism. A single ventricular septal defect, the absence of cardiac anomaly, or only abnormal ventricular looping can occur in patients with left-sided isomerism [1, 4, 6]. The gastrointestinal, hepatic, or renal system anomalies are seen in a considerably large spectrum. Solid intra-abdominal organs may be located in the midline, or it can be on its right or left side too [1, 5, 6]. Some problems such as atresia, agenesis, volvulus, or stenosis may be seen frequently [3]. The  presence of polysplenia or agenesis is used as an important classification parameter. Polysplenia describes the presence of a spleen either having more than one segment or consisting more than one segment. Gastrointestinal malrotation anomalies may appear in the form of a limited rotation or a reversed rotation in varying degrees [3].

There is a certain difficulty and risk in situs anomalies especially  in terms of diagnostic investigations and surgical operations [5]. Some of the cases may not meet criteria conforming a single classification, and it would be necessary to define the situation and the problem for a better patient management. When it comes to cardiac surgical procedures (biventricular repair and Fontan-type procedure) or extracardiac problems (biliary atresia, duodenal atresia, acute pancreatitis, appendicitis or cholecystitis, gastroschisis, etc.), a significant anatomical change for organ and vascular structures will increase the operative risk significantly [3, 5]. Therefore, setting forth the actual anatomy of the organs and vascular structures by using imaging modalities prior to a surgery or invasive intervention becomes important. In addition, BTFE-BH SENSE sequence on MRI seems to be useful in the evaluation of vascular variations such as renal vein variations [10].

In our literature research, we came across three extensive studies and several case presentations related to situs anomalies [1–8]. We compared the findings of our case with the findings of the situs ambiguous with polysplenia cases in those studies. Applegate et al. [1] reported a series of the ten polysplenic patients for heterotaxy syndrome. They showed that there were midline or left-sided liver in 6/10 patients, right-sided aortic arc and left-sided stomach and inferior vena cava in 4/10 patients, right-sided gallbladder and the absence of a congenital heart disease in 3/10 patients, left-sided polysplenia in 2/10 patients, and dextrocardia in only one of these patients. The findings of none of the patients in this group coincided one to one with the findings of the case that we reported here. Our case was different from the closest case of these series due to the right-sided cardiac apex, left-sided stomach, and the absence of congenital heart disease. Fulcher and Turner [2] reported eight adult cases for situs ambiguous with polysplenia. The characteristics of this patient group included midline/left-sided liver, left-sided stomach and left-sided polysplenia in 5/8 patients, right-sided gallbladder in 3/8 patients, and the right-sided cardiac apex, aorta, and inferior vena cava, small intestine and large intestine rotation anomaly absence, and normally-located pancreas in 1/8 patients. However, none of them had entire components forming the case we present here. Lee et al. [3] reported six cases of situs ambiguous with left isomerism. These patients had some intra-abdominal anomaly including malrotation in 4/6, biliary atresia in 1/6, and acute appendicitis in 1/6 of the cases. There was polysplenia in only one of them. The magnetic resonance imaging did not show the other types of anomalies except malrotation in our case. To our best knowledge, the case we present does not coincide one to one with any of the cases reported previously.

In conclusion, variations in situs ambiguous cases differ. Although the cases are tried to be classified in two main groups, variations are in a broad spectrum and a single description is not possible. It is crucial to reveal these variations by using imaging modalities because being aware of them prior to surgery and invasive intervention prevents the possible risks and complications.
